# Bilateral Internal Thoracic Artery and Optimal Revascularization Strategy in Insulin-Dependent Diabetic Patients

**DOI:** 10.5935/1678-9741.20150062

**Published:** 2015

**Authors:** Omar Asdrúbal Vilca Mejía, Luiz Augusto Ferreira Lisboa, Luís Alberto Oliveira Dallan, Fabio Biscegli Jatene

**Affiliations:** 1 Instituto do Coração do Hospital das Clínicas da Faculdade de Medicina da Universidade de São Paulo (InCor HC-FMUSP), São Paulo, SP, Brazil.

Coronary artery disease (CAD) is a leading cause of mortality and morbidity in diabetic patients^[1]^. Four among 10 patients undergoing coronary artery bypass graft surgery (CABG) are diabetic in the United States^[2]^. Furthermore, insulin-dependent diabetics undergoing CABG reaches 20% in São Paulo Registry of Cardiovascular Surgery (REPLICCAR) ^[3]^.

Currently, there is a progressive increase in the prevalence of diabetes in patients referred for CABG. This is mainly in response to FREDOOM trial that showed that diabetic patients have a higher survival rate when they undergo surgical myocardial revascularization^[4]^. Over time, one of the reasons that made the CABG overlap percutaneous techniques was the anastomosis of the internal thoracic artery (ITA) on the left anterior descending artery. Long-term benefits would be related to higher patency of the ITA graft compared to the saphenous vein graft^[5]^. Later studies in large databases were able to show that the use of bilateral internal thoracic artery (BITA) increased further patient survival^[6]^. In this respect, only one prospective and randomized study showed no difference in one year and awaited long term outcomes^[7]^.

Therefore, which could make us desist from using BITA? Demand for technical dissection of BITA and risk of deep sternal infection especially in diabetic patients would be the most frequent reasons. Gatti et al.^[8]^ showed that even in insulin-dependent diabetic patient advantagens with BITA is over. This means that the longer survival achieved with BITA is not affected by a higher incidence of deep sternal wound infection (DSWI) in the short term^[9]^. In order to justify the risk of this complication, patients should have a life expectancy of >10 years. Therefore, they are contraindicated for the most part of the time in diabetic individuals^[10]^.

CABG to insulin-dependent diabetic patient comprises various quality parameters. This begins with the classification in controlled and uncontrolled diabetics by glycosylated hemoglobin (HbA1c). It has been demonstrated that poorly controlled diabetes patients but not well-controlled diabetes significantly impairs endothelium-dependent and endothelium-independent relaxation of human peripheral microvasculature as compared with non-diabetes^[11]^. These changes may contribute to the less favorable postoperative outcomes after cardiac surgery. Complications and DSWI were significantly higher in uncontrolled (HbA1c >7%) than with controlled (<7%) diabetic patients^[11]^. Hyperglycemia in perioperative CABG increases up to 10 times the risk of morbidity and mortality. Thus, using protocols with continuous insulin drip to control hyperglycemia during the perioperative period (<180 mg/dl) reduced mortality, morbidity, incidence of DSWI, length of stay and increased long-term survival^[12]^.

Skeletonization technique instead of pedicle preparation confers a protective benefit against sternal wound infection in patients receiving BITA and this should be the technique of choice for diabetics in whom BITA harvest is desired^[13]^. Because of diffuse atherosclerotic disease and complex coronary anatomy, complete revascularization in CABG is recommended for diabetic patients^[14]^. Even more than that, the use of BITA plus incomplete revascularization is better than single AIT plus complete revascularization in a twenty-year-survival^[15]^. Thereby, BITA is the most important to CABG in diabetic patients.

With respect to CABG be performed with or without the use of cardiopulmonary bypass (CPB), a subanalysis of the ROOBY trial show higher rates of incomplete revascularization in diabetic patients operated with off-pump surgery^[16]^. In addition, the graft patency after 1 year was significantly lower in the off-pump CABG (83.1%) when compared to on-pump CABG (88.4%). Raza et al.^[15]^ shows that the hospital outcomes and long-term survival of patients undergoing off pump or on-pump surgery are similar.

The Guideline ESC / EACTS 2014 in CABG^[17]^ recommends that the use of BITA should be considered in patients <70 years (Class IIa). Also, it provides that BITA in diabetic patients should be considered. Thus, all diabetic patients under 70 years, without morbid obesity and HbA1c <7% should receive BITA. On the other hand, skeletonized dissection of ITAs is recommended in these patients. After this support, the use of BITA as a quality standard in insulin-dependent diabetic patients is advised after CABG.

However. it might be best to avoid BITA grafting in obese diabetic women with diffuse atherosclerotic, the use of BITA becomes the best cost-effectiveness revascularization strategy to long term survival even in insulin-dependent diabetic patients. This is reinforced by a growing incidence in coronary artery disease in young individuals and increase in life expectancy in developing countries. Therefore, underutilized BITA, 4.4% in the STS database^[18]^ and around 10% in the European Database^[19]^, contradict the recommendations given by the respective host societies. REPLICCAR showed better results, and it is the first multicenter prospective cohort of patients undergoing cardiovascular surgery in São Paulo that has 10% of BITA^[20]^.

In conclusion, BITA grafting is recommended for patients with diabetes undergoing CABG and efforts should be made to complete revascularization. DSWI, because of its rare occurrence, had little effect on morbidity and mortality increase. Accordingly, the state-of-the-art coronary artery bypass surgery in insulin-dependent diabetic patients considering the use of BITA is a quality parameter of health care.
